# Wound of the Throat

**Published:** 1829-04

**Authors:** 


### WOUND OF THE THROAT.

### *Wound of the Throat; Disease of the Larynx.*

(ST. GEORGE'S HOSPITAL.)

WM. GRENYON, æt. forty-six, admitted January 27th, under the care of Mr. HAWKINS, having in despondency inflicted a deep wound on the left side of the neck, from below the ear towards the front of the chin, the line of the wound being some way above the os hyoides. There had been hemorrhage, according to report, to the amount of a quart, and on his admission he was very faint from loss of blood.

He remained perfectly composed and free from fever till Feb. 5th, having only complained of a little cough, which commenced on the 31st. The wound at this time was granulating, and perfectly healthy.

Feb. 5th.—He was seized with great difficulty of breathing and swallowing, totally lost his voice, had expectoration of large quantities of thick tenacious mucus; his pulse became extremely rapid, but small and weak; his countenance had an expression of intense anxiety, and the larynx was tumid and tender. He had all the symptoms, in short, of acute laryngitis, in a debilitated constitution.

Twelve leeches were immediately applied to the larynx, which was afterwards fomented, and he was directed to inhale the steam of warm water constantly; and he took five grains of calomel and one and a half grain of opium directly.

Under this treatment the symptoms soon subsided, so that the next day (Feb. 6th,) his pulse was only 102, and perfectly quiet; and all difficulty of breathing had gone, though he was still unable to swallow with facility.—Ordered to continue the medicine he had previously taken; saline mixture with paregoric; and to take some syrup for his cough.

9th.—He had a return of laryngitis in a slighter degree, which was again relieved by twelve leeches, and a repetition of the same medicine.

11th.—Not much difficulty in breathing or swallowing; but some cough is always produced by deglutition, though not to the same degree as a few days since. He makes no complaint, except of the cough. There is no pain or tenderness of the throat, nor is there any pain in the chest; and he can inspire deeply and freely without any irritation of the lungs: yet within the last three days he has become evidently much worse. His countenance is sallow, the features shrunk, with an expression of great anxiety; his breathing is hurried, and with a rattling noise from the expectoration, which is very copious, and has a small quantity of pus mixed with the mucus. His pulse is above 120, irritable and weak; tongue clean, and generally moist. It was evident that he was sinking, and, as Mr. Hawkins judged, from irritation spreading downwards from the larynx, and probably producing effusion into the lungs; for the small quantity of purulent secretion and the absence of pain did not lead him to expect phthisis, though the patient said he had been subject to a cough before his admission.

14th.—Some slight tumefaction was observed just above the sternum, which led Mr. Hawkins to suspect that an abscess might have formed; and, on pressing deeply on the right side of the trachea, it appeared as if some fluid could be made to project by the side of the left sterno-cleido-mastoideus muscle, but too indistinct for any one present to decide that this was the case. There was not the slightest pain or tenderness on pressure, nor did pressing this part produce any difficulty of breathing; and the trachea could easily be felt below the integuments; so that it was evident, if matter did exist, that it did not produce pressure on the trachea. Even this slight swelling was less distinct when the throat was examined the next day, during which he sunk gradually, and died in the afternoon.

*Dissection.*—A considerable swelling was now perceptible in the fore part of the throat, in which the fluctuation of fluid was very evident; and, on dissection, several ounces of thick pus were found in front of the larynx and trachea, reaching from the os hyoides to the sternum, behind which the matter was in contact with the deep cervical fascia. The surface of the thyroid and cricoid cartilage, and of the trachea, was quite exposed, the covering of the abscess being formed anteriorly by all the muscles below the os hyoides except the crico thyroideus, which remained at the bottom of the abscess. By the contraction of those muscles the abscess had been made to extend itself principally by the side of the trachea, under the sterno-cleido-mastoidei muscles, and below the sternum towards the chest; and had thus been prevented from projecting in front of the trachea during life. The parts between the bottom of the wound and the commencement of the abscess were perfectly sound.

On opening the larynx, the soft parts between the epiglottis and the superior thyro-arytænoid ligament, on each side, were very much thickened by the deposition of lymph beneath the mucous membrane; and a small ulcer was found at the mouth of the epiglottis. Just above this ligament, on the right side, a small opening led into a cavity completely surrounding the arytænoid cartilage, portions of which had exfoliated and were loose in the cavity; and nearly the whole of the cartilage was laid bare, the joint alone being healthy. The greater part of all the cartilages were converted into bone. The inner membrane of all the air tubes was highly turgid with vessels; and both lungs contained more mucus than usual, especially the right, the lower part of which was condensed and heavy, from the quantity which had been secreted; and a few flakes of recent lymph were on the surface of the pleura. The other viscera were healthy.

The disease in the larynx must, no doubt, have existed for some time before he inflicted the wound in the throat, as ossification and exfoliation are comparatively slow processes; but the coincidence with a wound in the neighbourhood is curious, and contributed to produce the obscurity in the progress of the symptoms. The wound may, perhaps, have accelerated the disease, though it was not likely to be the immediate cause of the abscess, from the healthy state of the granulations and the gradual filling up of the incision. The abscess being directly in contact with the membrane of the larynx and trachea, and the pus being in equal quantity on both sides of the larynx, (or, if any thing, more copious on the right side,) were sufficient, in Mr. Hawkins's opinion, to show that it had been the consequence of the inflammatory condition of the inner membrane of the tube, and the irritation of the internal abscess round the right arytænoid cartilage, from which the purulent expectoration had proceeded during life.

